# Real-World Effectiveness of Finerenone Added to SGLT2 Inhibitor and GLP-1 Receptor Agonist Therapy in Individuals with Type 2 Diabetes and Chronic Kidney Disease

**DOI:** 10.3390/jcm14228209

**Published:** 2025-11-19

**Authors:** Afif Nakhleh, Khaled Khazim, Naim Shehadeh

**Affiliations:** 1 Maccabi Healthcare Services, Haifa 3299001, Israel; khazim_kh@mac.org.il (K.K.); naim.shehadeh@biu.ac.il (N.S.); 2 The Azrieli Faculty of Medicine, Bar-Ilan University, Safed 1311502, Israel

**Keywords:** chronic kidney disease, finerenone, glucagon-like peptide-1 receptor agonist, real-world study, sodium-glucose cotransporter-2 inhibitor, type 2 diabetes

## Abstract

**Background/Objectives:** Recent randomized controlled trial evidence in adults with type 2 diabetes (T2D) and chronic kidney disease (CKD) indicates that adding finerenone to empagliflozin provides additive clinical benefit. A prespecified analysis demonstrates that this benefit is consistent irrespective of prior glucagon-like peptide-1 receptor agonist (GLP-1 RA) use. We aimed to assess the effectiveness of adding finerenone to existing sodium-glucose cotransporter-2 inhibitor (SGLT2i) and GLP-1 RA therapy in a real-world setting. **Methods:** We performed a retrospective cohort study of adults with T2D and CKD from Maccabi Healthcare Services diabetes, endocrinology, and nephrology clinics in Haifa, Israel. Included individuals initiated finerenone between 1 August 2023, and 31 January 2025, and met the following criteria: estimated glomerular filtration rate (eGFR) of 25–60 mL/min/1.73 m^2^; urinary albumin-to-creatinine ratio (UACR) > 300 mg/g; and a history of ≥12 weeks of SGLT2i (empagliflozin or dapagliflozin) and GLP-1 RA (liraglutide, dulaglutide, or semaglutide) use prior to finerenone initiation. Outcomes were assessed at the last measurement taken within 26 ± 10 weeks of finerenone initiation. The primary outcome was adjusted percent change in log-transformed UACR from baseline to follow-up. Secondary outcomes were adjusted mean changes in eGFR and serum potassium. We used multiple linear regression models. Prespecified subgroup analyses examined the UACR change by age, sex, body mass index (BMI), baseline eGFR, and baseline UACR. **Results:** Fifty-one individuals were included in the study, with a mean age of 66.0 ± 9.5 years and a mean BMI 30.9 ± 5.2 kg/m^2^. The median eGFR was 45 mL/min/1.73 m^2^ (IQR 36–52), and the median UACR was 1001 mg/g (IQR 515–1599). 94% were receiving a renin–angiotensin system inhibitor. Finerenone was initiated at 10 mg/day and titrated to 20 mg/day in eight individuals. Over a median follow-up of 27 weeks, the adjusted percent change in UACR was −51.3% (*p* < 0.001), consistent across prespecified subgroups. The adjusted mean eGFR change was −3.92 mL/min/1.73 m^2^ (*p* < 0.001). Serum potassium increased by +0.34 mmol/L (*p* < 0.001). **Conclusions:** In adults with T2D and albuminuric CKD already receiving an SGLT2i and a GLP-1 RA, adding finerenone substantially reduced albuminuria.

## 1. Introduction

Chronic kidney disease (CKD) is a major global public health concern. It is estimated that one in ten adults in Europe, Canada, and Israel likely have CKD [1]. Diabetic kidney disease (DKD) stands as the primary cause of CKD globally, affecting approximately half of all patients with type 2 diabetes (T2D) [2]. Increasing albuminuria is associated with a higher risk of progressing to end-stage renal disease and increased mortality [3]. In addition to renin-angiotensin system (RAS) inhibitors, sodium-glucose cotransporter-2 inhibitors (SGLT2is), finerenone, a nonsteroidal mineralocorticoid receptor antagonist (MRA) and semaglutide, a glucagon-like peptide-1 receptor agonist (GLP-1 RA), have more recently been shown to reduce DKD progression and are considered the standard of care [4,5].

In the FLOW trial, semaglutide, a GLP-1 RA, reduced the risk of major kidney outcomes by 24% in individuals with T2D and CKD [6]. This benefit was consistent across all CKD severity categories defined by estimated glomerular filtration rate (eGFR) or albuminuria, and was accompanied by clinically relevant improvements in glycemic control, body weight, and blood pressure at 104 weeks [6].

In the CONFIDENCE trial, finerenone and empagliflozin combination therapy demonstrated an additive benefit for individuals with T2D and CKD (mean eGFR 54.2 mL/min/1.73 m^2^, median urinary albumin-to-creatinine ratio (UACR) 579 mg/g). After 180 days, UACR reductions were significantly greater with the combination therapy (29% more than finerenone and 32% more than empagliflozin) [7]. A recent prespecified analysis of the CONFIDENCE trial evaluated whether the efficacy and safety of finerenone and empagliflozin combination therapy varied by baseline GLP-1 RA use [8]. Among 800 adults with T2D and CKD, 182 (23%) were GLP-1 RA users at baseline. Of these, 68 (8.5%) participants initiated combination therapy. At 180 days, GLP-1 RA users receiving the combination had a 51% reduction in UACR (compared to 34% with finerenone alone and 36% with SGLT2i alone) [8]. This effect mirrored results seen in non-users of GLP-1 RA, with similar eGFR trajectories and hyperkalemia rates across both strata.

Given these findings, the effect of adding finerenone to existing dual therapy with an SGLT2i and a GLP-1 RA in individuals with T2D and CKD warrants real-world observational evaluation The aim of this study was to assess the impact of adding finerenone to existing SGLT2i and GLP-1 RA therapy by examining adjusted percent change in UACR and adjusted changes in eGFR and serum potassium after finerenone initiation.

## 2. Materials and Methods

We conducted this retrospective cohort study using de-identified electronic records from Maccabi Healthcare Services (MHS), Israel’s second-largest health maintenance organization.

### 2.1. Study Subjects and Definitions

**Figure 1:**
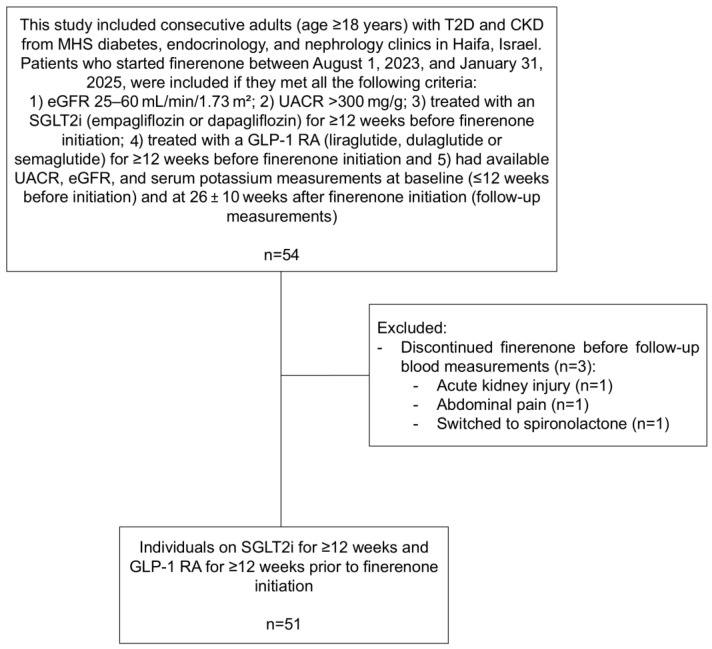
Flow diagram of individuals included in the study. Abbreviations: CKD, chronic kidney disease; eGFR, estimated glomerular filtration rate; GLP-1 RA, glucagon-like peptide-1 receptor agonist; MHS, Maccabi Healthcare Services; SGLT2i, sodium-glucose co-transporter 2 inhibitor; T2D, type 2 diabetes; UACR, urinary albumin-to-creatinine ratio.

We included adults (≥18 years) with T2D and CKD from MHS diabetes, endocrinology, and nephrology clinics in Haifa, Israel, between 1 August 2023, and 31 January 2025, who met all the following criteria: (1) eGFR 25–60 mL/min/1.73 m^2^; (2) UACR > 300 mg/g; (3) treated with an SGLT2i (empagliflozin or dapagliflozin) for ≥12 weeks before finerenone initiation; (4) treated with a GLP-1 RA (liraglutide, dulaglutide or semaglutide) for ≥12 weeks before finerenone initiation and (5) had available UACR, eGFR, and serum potassium measurements at baseline (≤12 weeks before initiation) and at 26 ± 10 weeks after finerenone initiation (follow-up measurements). Follow-up concluded on the date of the final UACR measurement recorded within 26 ± 10 weeks of finerenone initiation. Only individuals with eGFR and serum potassium measurements within this same timeframe were included, with the last recorded value used for each parameter. We excluded individuals who discontinued finerenone before follow-up blood measurements at 26 ± 10 weeks (Figure 1).

Data were community-based clinical and laboratory measures. All labs were analyzed at an MHS-certified central laboratory. Diagnoses and medications were identified using International Classification of Diseases, 9th Revision, Clinical Modification (ICD-9-CM) and Anatomical Therapeutic Chemical (ATC) codes. eGFR was calculated using the 2021 CKD-EPI equation [4].

### 2.2. Study Outcomes

The primary outcome was the adjusted percent change in UACR from baseline to follow-up (estimated from a model using UACR). Secondary outcomes were adjusted changes in eGFR and serum potassium.

### 2.3. Statistical Analysis

We used a multiple linear regression for the primary model, adjusting for baseline UACR, age, sex, baseline body mass index (BMI), baseline glycated hemoglobin (HbA1c), and baseline eGFR. We report the least-squares mean ratios with 95% confidence intervals (CIs) and converted them to a mean adjusted percentage change using the formula: 100 × (ratio − 1). For the secondary models, we used multiple linear regression to estimate adjusted mean changes. For eGFR, the model was adjusted for baseline eGFR, age, sex, baseline BMI, and baseline HbA1c. For potassium, it was adjusted for baseline potassium, age, sex, and baseline eGFR. Prespecified subgroup analyses assessed percent UACR change by sex, age (<65 vs. ≥65), BMI (<30 vs. ≥30 kg/m^2^), baseline eGFR (<45 vs. ≥45 mL/min/1.73 m^2^), and baseline UACR (<1000 vs. ≥1000 mg/g) using interaction terms. Analyses were performed using SAS version 9.4 (SAS Institute Inc., Cary, NC, USA).

## 3. Results

Of 54 individuals, 51 fulfilled all inclusion criteria (Figure 1).

Baseline characteristics are detailed in Table 1. The cohort had a mean age of 66.0 ± 9.5 years, mean BMI of 30.9 ± 5.2 kg/m^2^, and mean HbA1c of 7.2 ± 1.3%. Baseline median eGFR was 45 mL/min/1.73 m^2^ (IQR 36–52), and median UACR was 1001 mg/g (IQR 515–1599). A total of 48 (94%) individuals were treated with a RAS inhibitor. Before starting finerenone, these individuals had been treated with a GLP-1 RA for a median of 28 months (IQR 12–38) and an SGLT2i for a median of 30 months (IQR 17–44). Finerenone was initiated at a dose of 10 mg/day and titrated to 20 mg/day in eight individuals during follow-up.

After a median of 27 weeks (IQR 21–30) of finerenone treatment, the median UACR was 421 mg/g (IQR 232–923). Overall, 35/51 (68.6%) patients achieved a ≥30% reduction in UACR, and 25/51 (49.0%) achieved a ≥50% reduction. The adjusted percentage change in UACR was a 51.3% reduction (least-squares mean ratio 0.487; 95% CI 0.386, 0.616; *p* < 0.001) (Table 2).

**Figure 2:**
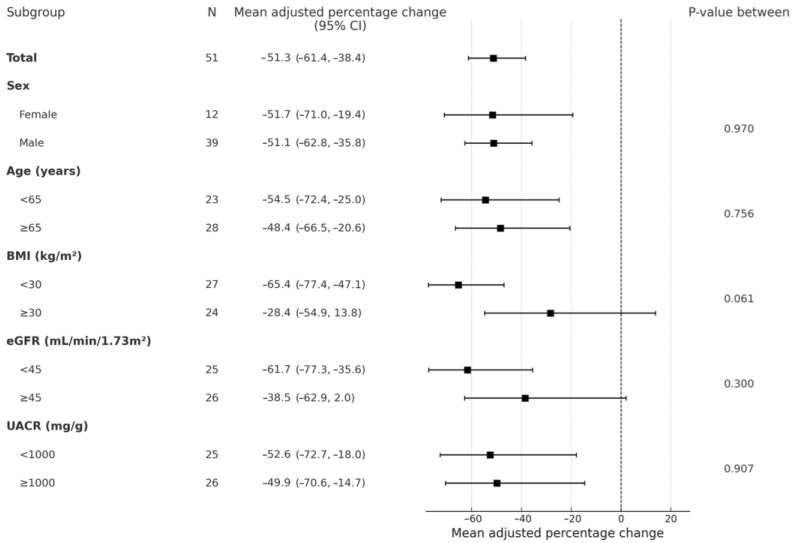
Forest plot of change in mean adjusted UACR from baseline to follow-up, showing prespecified subgroup analyses. Abbreviations: BMI, body mass index; eGFR, estimated glomerular filtration rate; UACR, urinary albumin-to-creatinine ratio.

In subgroup analyses, this reduction was consistent across prespecified subgroups (Figure 2). At follow-up, the median eGFR was 39 mL/min/1.73 m^2^ (IQR 32–48), and the adjusted mean change in eGFR was −3.92 mL/min/1.73 m^2^ (95% CI −5.79, −2.05; *p* < 0.001) (Table 2). The median serum potassium was 4.9 mmol/L (IQR 4.6–5.1), and the adjusted mean change was +0.34 mmol/L (95% CI +0.22, +0.47; *p* < 0.001) (Table 2). Two individuals had serum potassium >5.5 mmol/L and were successfully managed with potassium binders.

## 4. Discussion

To our knowledge, this is the first real-world study to assess the effect of adding finerenone to SGLT2i and GLP-1 RA therapy in adults with T2D and CKD. This addition resulted in a ~51% reduction in UACR over approximately six months, a finding that was consistent across subgroups (including baseline eGFR and UACR). The magnitude of albuminuria lowering aligns with the 52% reduction reported in the finerenone and empagliflozin combination arm of the CONFIDENCE trial [7]. It also mirrors the 51% reduction in UACR observed among the 68 individuals who received finerenone and empagliflozin and were on a GLP-1 RA at baseline in a recent analysis of the CONFIDENCE trial [8].

In contrast to the CONFIDENCE trial, where finerenone and empagliflozin were initiated simultaneously, all individuals in our study had been receiving an SGLT2i (median, 30 months) and a GLP-1 RA (median, 28 months) before finerenone was added. Despite having more advanced kidney disease and higher baseline albuminuria than participants in CONFIDENCE, our cohort showed a robust reduction in albuminuria after finerenone initiation. These findings suggest that finerenone is effective across disease severity and treatment sequences, with its effect on albuminuria-lowering maintained even when added to established SGLT2i and GLP-1 RA therapy.

The adjusted eGFR decline (−3.92 mL/min/1.73 m^2^) may reflect a hemodynamic dip. This value is close in magnitude to the −5.0 mL/min/1.73 m^2^ adjusted eGFR decline observed in the combination therapy group of the CONFIDENCE trial at day 180, which largely stabilized thereafter [7]. Importantly, in the CONFIDENCE trial most of the early eGFR decline was reversible after treatment discontinuation, supporting the interpretation that this is a functional, drug-related effect rather than progressive renal impairment. Nevertheless, longer follow-up is needed to determine the full trajectory. Of note, one of the three excluded individuals had acute kidney injury during the first month after initiating finerenone (Figure 1). This uncommon incidence is consistent with the low incidence of acute kidney injury observed in the combination therapy group in the CONFIDENCE trial (~2%) [7]. The modest increase in potassium (+0.34 mmol/L) supports the need for routine monitoring yet suggests acceptable short-term tolerability, with only two individuals >5.5 mmol/L who were managed using potassium binders.

In light of recent evidence, a four-pillar strategy including RAS blockade, SGLT2 inhibition, nonsteroidal MRA, and GLP-1 RA is a well-founded approach for patients with T2D and albuminuric CKD. This approach leverages complementary renoprotective mechanisms. RAS blockade provides favorable hemodynamic effects. Beyond their glucose-lowering properties, SGLT2is may exert pleiotropic effects and provide both hemodynamic and metabolic benefits, thereby enhancing cell survival and function under stress conditions [4,9]; GLP-1 RAs target metabolic and inflammatory drivers; and the nonsteroidal MRA, finerenone, adds hemodynamic, anti-fibrotic, and anti-inflammatory benefits [10]. Renal injury in CKD involves mechanisms in which mineralocorticoid receptor (MR) overactivation, elevated fibroblast growth factor 23 (FGF23), and Klotho insufficiency act together to accelerate kidney damage. MR activation promotes intrarenal inflammation, oxidative stress, and fibrosis, while Klotho deficiency and excess FGF23 further amplify these pathways. By selectively antagonizing MR, finerenone may attenuate both the direct MR-mediated renal injury and the intersecting FGF23/Klotho-related cascades, leading to beneficial renal effects [11]. Furthermore, experimental data indicate that finerenone can even reverse diabetes-related downregulation of renal GLP-1 receptors, providing a strong mechanistic rationale for combining finerenone with GLP-1 RAs [12].

The study has several limitations, including its single-center, uncontrolled design and relatively small sample size. We could not separately analyze the effect of the 10 mg versus 20 mg dose due to the study’s design and small sample size. A further limitation is that we did not adjust our models for every individual background antihypertensive drug class. The vast majority of participants (94%) were already receiving RAS inhibitor therapy. We therefore treated its effect as essentially constant across the cohort and focused on the additive effect of finerenone. Other agents such as beta blockers (75%) and calcium channel blockers (63%) were also common, but these classes are not typically associated with albuminuria reductions of the magnitude we observed [13]. Adding all of these medication variables separately to our model would have increased the risk of overfitting and reduced the precision of the main estimate. We assume that any residual confounding from these medications is likely to be small and does not change the conclusion that finerenone provided a substantial additive reduction in UACR. Additionally, due to its observational nature, confounding by unmeasured factors cannot be excluded.

## 5. Conclusions

The study provides pragmatic, real-world evidence that adding finerenone to established SGLT2i and GLP-1 RA therapies yields a large (~51%) reduction in albuminuria within approximately 6 months, accompanied by a modest dip in eGFR and manageable increases in serum potassium levels. The consistency across prespecified subgroups and the similarity in magnitude to effects observed in randomized trials reinforce confidence in this signal. These findings support implementing a “four-pillar” approach in routine care for patients with T2D and albuminuric CKD. Larger studies with longer follow-up are needed to confirm durability and clinical outcome benefits, including among patients with lower-grade albuminuria.

## Figures and Tables

**Table 1:** Baseline characteristics of the study group. Abbreviations: BMI, body mass index; eGFR, estimated glomerular filtration rate, GLP-1 RA, glucagon-like peptide-1 receptor agonist; HbA1c, glycated hemoglobin; RAS, renin-angiotensin system; SD, standard deviation; SGLT2i, sodium-glucose co-transporter 2 inhibitor; UACR, urinary albumin-to-creatinine ratio.

Characteristic	SGLT2i plus GLP-1 RA n = 51
Age, mean (SD), years	66.0 (9.5)
Male, n (%)	39 (76)
BMI, mean (SD), kg/m^2^	30.9 (5.2)
HbA1c, mean (SD), %	7.2 (1.3)
UACR, median (IQR), mg/g	1001 (515–1599)
eGFR, median (IQR), mL/min/1.73 m^2^	45 (36–52)
Potassium, median (IQR), mmol/L	4.6 (4.4–4.7)
Hypertension, n (%)	51 (100)
Atherosclerotic cardiovascular disease, n (%)	20 (39)
Chronic heart failure, n (%)	11 (22)
Hyperlipidemia, n (%)	49 (96)
RAS inhibitor, n (%)	48 (94)
Diuretic, n (%)	9 (18)
Potassium binder, n (%)	3 (6)
Beta blocker, n (%)	38 (75)
Calcium channel blocker, n (%)	32 (63)
Insulin, n (%)	28 (55)
Metformin, n (%)	27 (53)
Statin, n (%)	48 (94)
Aspirin, n (%)	23 (45)
Clopidogrel, n (%)	4 (8)

**Table 2:** Multivariable models for primary and secondary outcomes.

Outcome *	Parameter (Finerenone Follow-Up vs. Baseline)	Effect Estimate	95% CI	*p*-Value
UACR ^†^ (log-transformed)	LS mean ratio	0.487	0.386, 0.616	<0.001
eGFR ^‡^ (mL/min/1.73 m^2^)	Adjusted mean change	−3.92	−5.79, −2.05	<0.001
Serum potassium ^§^ (mmol/L)	Adjusted mean change	+0.34	+0.22, +0.47	<0.001

^†^ Model adjusted for baseline log-UACR, age, sex, BMI, HbA1c, eGFR. ^‡^ Model adjusted for baseline eGFR, age, sex, BMI, HbA1c. ^§^ Model adjusted for baseline potassium, age, sex, baseline eGFR. * All models were assessed for multicollinearity using variance inflation factors (VIF), and all VIFs were <2, indicating no meaningful multicollinearity among included predictors. Abbreviations: CI, confidence interval; BMI, body mass index; eGFR, estimated glomerular filtration rate; HbA1c, glycated hemoglobin; UACR, urinary albumin-to-creatinine ratio.

## Data Availability

The data presented in this study are available on request from the corresponding author.

## Abbreviations

The following abbreviations are used in this manuscript:

ATC	Anatomical Therapeutic Chemical
BMI	Body mass index
CI	Confidence interval
CKD	Chronic kidney disease
CKD-EPI	Chronic Kidney Disease Epidemiology Collaboration
CONFIDENCE	Combination effect of finerenone and empagliflozin
DKD	Diabetic kidney disease
eGFR	Estimated glomerular filtration rate
FLOW	Evaluate renal function with semaglutide once weekly
GLP-1 RA	Glucagon-like peptide-1 receptor agonist
HbA1c	Glycated hemoglobin
ICD-9-CM	International Classification of Diseases, 9th Revision, Clinical Modification
IQR	Interquartile range
MHS	Maccabi Healthcare Services
MRA	Mineralocorticoid receptor antagonist
RAS	Renin–angiotensin system
SGLT2i	Sodium–glucose cotransporter-2 inhibitor
T2D	Type 2 diabetes
UACR	Urinary albumin-to-creatinine ratio
